# The Role of Genetic Polymorphisms in Differentiated Thyroid Cancer: A 2023 Update

**DOI:** 10.3390/biomedicines11041075

**Published:** 2023-04-02

**Authors:** Robert Aurelian Tiucă, Oana Mirela Tiucă, Ionela Maria Pașcanu

**Affiliations:** 1 Doctoral School of Medicine and Pharmacy, George Emil Palade University of Medicine, Pharmacy, Sciences and Technology of Targu Mures, 540142 Targu Mures, Romania; 2 Department of Endocrinology, George Emil Palade University of Medicine, Pharmacy, Sciences and Technology of Targu Mures, 540142 Targu Mures, Romania; 3 Compartment of Endocrinology, Mures County Clinical Hospital, 540139 Targu Mures, Romania; 4 Department of Dermatology, George Emil Palade University of Medicine, Pharmacy, Sciences and Technology of Targu Mures, 540142 Targu Mures, Romania; 5 Dermatology Clinic, Mures County Clinical Hospital, 540015 Targu Mures, Romania

**Keywords:** thyroid cancer, differentiated thyroid cancer, thyroid cancer prognosis, molecular biomarkers, targeted therapy, single nucleotide polymorphism, genetic variation, genetic predisposition

## Abstract

Thyroid cancer is the most common endocrine malignancy, with an increasing trend in the past decades. It has a variety of different histological subtypes, the most frequent one being differentiated thyroid cancer, which refers to papillary carcinoma, the most common histological type, followed by follicular carcinoma. Associations between genetic polymorphisms and thyroid cancer have been investigated over the years and are an intriguing topic for the scientific world. To date, the results of associations of single nucleotide polymorphisms, the most common genetic variations in the genome, with thyroid cancer have been inconsistent, but many promising results could potentially influence future research toward developing new targeted therapies and new prognostic biomarkers, thus consolidating a more personalized management for these patients. This review focuses on emphasizing the existing literature data regarding genetic polymorphisms investigated for their potential association with differentiated thyroid cancer and highlights the opportunity of using genetic variations as biomarkers of diagnosis and prognosis for thyroid cancer patients.

## 1. Introduction

**Figure 1:**
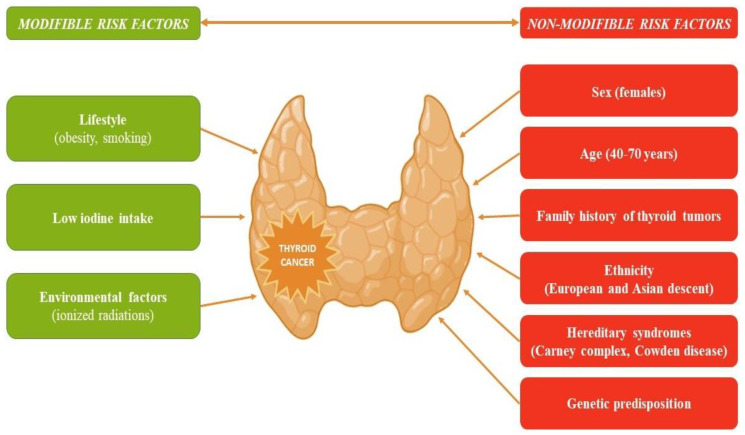
Risk factors that increase the likelihood of developing thyroid cancer.

Thyroid cancer, the most frequent endocrine malignancy, has recorded an increase in global incidence in recent decades. This increase is mostly attributed to the increased detection of low-risk tumors through intensive screening, which may result in an overdiagnosing phenomenon [1]. Papillary thyroid carcinoma (PTC) is the most common histological type, followed by follicular thyroid carcinoma (FTC). The papillary and follicular histological types are known as differentiated thyroid cancer (DTC) to distinguish them from poorly differentiated thyroid and anaplastic carcinomas, which originate from the follicular tissue, as well as from medullary thyroid cancer, which is derived from the parafollicular C cells [2]. Several risk factors, modifiable and non-modifiable, have been linked to an increased likelihood of developing thyroid cancer (Figure 1) [3].

The prognosis in DTC is often favorable, but it is influenced by the tumor’s histological type. PTC has an excellent prognosis, although a small proportion of patients show an aggressive, unfavorable evolution [4,5]. In recent years, molecular markers such as B-Raf Proto-Oncogene, Serine/Threonine Kinase (*BRAF*) V600E and Neuroblastoma RAS viral oncogene homolog (*NRAS*) mutations, and more recently, mutations in telomerase reverse transcriptase promoter (*pTERT*), have been studied for their role as predictors in treatment response, as well as their association with various clinico-pathological features, recurrence, and mortality in patients with DTC [6,7]. The co-existence of *pTERT* and the *BRAF* V600E mutation is associated with an unfavorable prognosis, distant metastases, and increased mortality [8,9]. As opposed to genetic mutation, which is defined as any abnormal change in a DNA sequence, a genetic polymorphism is a DNA sequence variation commonly found in the population [10]. 

The identification of patients diagnosed with thyroid cancer at risk of having an unfavorable prognosis has great importance in optimizing individual therapeutic management. This narrative review aims to emphasize the existing literature data regarding genetic polymorphisms investigated for their potential association with DTC and highlight the opportunity of using genetic variations as biomarkers of diagnosis and prognosis for thyroid cancer patients.

## 2. Methodology

Relevant studies published between 2001 and 2023 were identified via PubMed search using various combinations of keywords such as “thyroid cancer”, “differentiated thyroid cancer”, “genetic polymorphism”, “single nucleotide polymorphism”, and “prognosis”. Furthermore, additional articles were identified by reviewing the reference list of important publications. All reviewed papers were written in English and data relevant to this article’s topic were extracted.

## 3. Single Nucleotide Polymorphism and Thyroid Cancer

The data obtained following genetic analysis might have a great impact on the evolution, prognosis, and treatment of several diseases, particularly cancer given the immense diversity of genetic alterations usually associated with it. Identifying patients with DTC prone to have a poor prognosis has crucial importance in optimizing individualized therapeutic management. It is widely known that epigenetic alterations such as DNA methylation, chromatin remodeling, histone modification, or noncoding RNA regulation are quite commonly seen in thyroid cancers [11]. Nucleotide changes in various gene locations may lead to carcinogenesis by either the overactivation of oncogenes or by silencing the suppressor genes. Germline mutations occur familiarly, therefore they are inherited, and they can modify the genomic profile, increasing the risk of future cancers, such as thyroid cancer [12,13,14]. 

Among genetic variations, single nucleotide polymorphism (SNP) is the most common. An SNP hosts a variation in the DNA sequence involving a single nucleotide. The mechanism behind these SNPs usually involves point mutations/substitutions, deletions, insertions, or base-pair multiple repeats [12]. Determining SNPs is a facile and common way to detect genetic variation, making them an easy target for genetic research. In general, an SNP is considered “common” if its frequency is ≥1% in the general population; below this value it is considered “rare”. Some authors rate SNPs as “common” at a frequency ≥5% in the general population, “rare” at a frequency between 1 and 5%, and “subpolymorphic” at a frequency ≤1% [15,16]. Frequently, SNPs have a “benign” nature, without a significant impact on the corresponding genes, enhancing the human population diversity. On the other hand, if serious nucleotide changes do happen, events such as aberrant enzymatic activity and abnormal biochemical reactions might occur, even a shift towards a “malignant” behavior, leading to carcinogenesis [17,18,19]. In contrast to polymorphisms that are relatively common in the human population, mutations represent an abnormal, permanent change following a lesion at the level of the DNA structure in a particular organism, occurring in less than 1% of the population. More precisely, mutation refers to an allelic variant prevalent in the general population undergoing mutational events that change the allele into a rare or abnormal allele. The human genome suffers multiple and varied injuries. Still, the rate of mutations remains relatively low due to the intervention of DNA repair mechanisms that recognize the damage that has occurred in the DNA structure [10].

The extent of a genetic component in any form of cancer can be estimated if a family-cancer database is available in a country. Sweden has such a database, and according to it, thyroid cancer registered the highest susceptibility accounted for by genetic effects (53%) [20]. Kerber et al., in a nested case-control study of the familial risk of 40 cancers conducted on a cohort of 662.515 individuals, reported that thyroid cancer presented a population attributable risk due to familial factors of 28% [21]. The main aim of investigating genetic associations is the identification of genetic variations that correlate with quantitative traits (such as height, weight, blood pressure, or behavior) or with a specific phenotype (such as cancer). 

Over the years, research regarding the association between genetic variation and cancer risk has been an intriguing topic for the scientific community given the opportunity to develop a personalized treatment with a more targeted therapy based on the genomic profile. Several studies in the literature have investigated the relationship between SNPs in various genes and DTC, although the results are often difficult to interpret due to inconsistent results.

### 3.1. SNPs of Tyrosine-Kinase Family Genes

#### 3.1.1. SNPs of *RET* Gene

The Rearranged during Transfection (*RET*) protooncogene encodes a receptor of the tyrosine-kinase proteins and has been associated with numerous types of cancers, including thyroid cancer. The process leading to carcinogenesis implies gain-of-function mutations that translate to *RET* activation [22]. Santos et al. conducted a case-control study in 2014 on a total of 1088 individuals (545 with DTC and 543 controls) to determine the effect of four SNPs (G691S, L769L, S836S, and S904S) of *RET* in the risk for DTC. It was noted that *RET* S836S is overexpressed in patients with PTC as well as the GGTC haplotype, suggesting that it might be a risk factor for PTC. Interestingly, the overrepresentation of minor alleles G691S and S904S was identified in an almost significant percentage of tumors >10 mm at diagnosis, hinting at a possible role in tumor behavior. No such associations were found in FTC [23]. On the other hand, He et al. noted that the haplotype CGGATAA of rs17028, rs1799939, rs1800858, rs1800860, rs2075912, rs2565200, and rs2742240 was associated with a reduced risk of DTC (OR = 0.18, *p* = 0.001) in a candidate gene association study conducted on 552 subjects (300 with DTC and 252 controls). In the same study, rs1799939 AG or AG plus AA genotypes were associated with increased risk for DTC without concomitant thyroid benign disorders (OR = 1.93, *p* = 0.009 and OR = 1.88, *p* = 0.011, respectively). Moreover, an increased risk for distant metastases was found for haplotype CAAGCGT of rs17028, rs1799939, rs1800858, rs1800860, rs2075912, rs2565200, and rs2742240 (OR = 7.57, *p* = 0.009) [24]. 

Although this research offers interesting information, the study population involved in both studies was relatively small, therefore the impact of *RET* SNPs on DTC risk might be underestimated. Furthermore, neither one of the studies assessed radiation exposure, a factor that has a great impact on the oncogenesis of thyroid malignancy. A recent meta-analysis showed strong epidemiological evidence of associations through the Venice criteria and false-positive report probability between thyroid cancer susceptibility and *RET* rs1799939 [25].

#### 3.1.2. SNPs of *MET* Gene

The cellular mesenchymal-epithelial transition (MET) factor is a plasma membrane tyrosine kinase receptor with low activity in normal cells, which may become activated in tumor cells through mutations, amplifications, or overexpression, leading to a potential increase in the aggressiveness of cancer [26,27]. The role of *MET* gene SNPs in PTC was investigated by Ning et al., who evaluated 858 patients with PTC [28]. *MET* SNP (rs1621) showed a significant association with PTC in females. Moreover, the rs1621 AG genotype might be especially associated with the female sex as it was significantly higher in the PTC group for female patients and had an increased risk of PTC. On the other hand, the analyzed *MET* SNPs revealed no correlation with metastasis and prognosis, regardless of gender [28]. 

### 3.2. SNPs of Genes Involved in Apoptosis, Genome Stability, and DNA Repair

#### 3.2.1. SNPs of *BAX* Gene

B-cell lymphoma 2-associated X protein (BAX) has an important role in mitochondrial apoptosis [29]. There might be an association between *BAX* gene polymorphism and oncogenesis, mainly due to the −248 G > A polymorphism that down-regulates the *BAX* gene transcription which ultimately might inhibit the apoptosis in tumoral cells [30]. However, a meta-analysis from 2013 suggested that *BAX* −248 G > A polymorphism is not to be considered an important oncogenic factor [31]. As for research which assessed this polymorphism and DTC, Cardoso-Duarte et al. recently conducted a case-control study on 30 Brazilian patients with PTC and concluded that the *BAX* single nucleotide polymorphism −248 G > A GG genotype was associated with PTC and the presence of the G allele was a protective factor against the occurrence of PTC [32].

#### 3.2.2. SNPs of *TP53* and *p21* Gene

It is well known that events leading to DNA damage critically raise the risk of developing cancer. Tumor protein 53 (TP53) has a main role in protecting the genome from getting damaged; therefore, it plays an important part in cancer development, including thyroid cancer [33]. Depending on the severity of DNA damage, TP53 determines if the cell undergoes a repairing process or apoptosis [34]. The p21 protein is a cyclin-dependent kinase inhibitor that is expressed by the activated TP53 during the G1 phase of the cell cycle, inhibiting DNA replication and therefore stopping the progression of the cell cycle [35]. Therefore, there are data stating that in more than half of all cancers, TP53/p21 is inactivated [36]. 

Heidari et al. investigated if SNPs in *TP53* (rs1042522) and *p21* (rs1059234 and rs1801270) genes affect the risk of PTC or if they are associated with clinical and histopathological features of PTC. The study was designed as a case-control study and found that the *TP53*-rs1042522 CC genotype was significantly associated with protection against PTC in the dominant, recessive, and allelic models (*p* = 0.008, *p* = 0.01, *p* = 0.002, respectively). Moreover, the rs1042522 was associated with tumors > 1 cm in dominant and recessive models (*p* = 0.04, *p* = 0.009, respectively) and with vascular invasion in the dominant model (*p* = 0.01). However, regarding SNP of the *p21* gene (rs10559234 and rs1801270), no correlation was found with the risk of PTC or clinical and histopathologic features [37]. There is little data in the literature concerning SNP in these genes and their role in thyroid cancer; therefore, there is a need to expand the research towards this area knowing the role they have in suppressing carcinogenesis. 

#### 3.2.3. SNPs of *HOTAIR* Gene

SNPs in the HOX Transcript Antisense RNA (*HOTAIR*) gene, which is an oncogene that regulates gene expression and chromatin dynamics, have been associated with breast and colorectal cancers [38,39]. Regarding thyroid cancer, there are mixed results depending on the SNP studied. Rad et al. found that *HOTAIR* rs1899663 gene polymorphism was not associated with clinical or histopathological features of thyroid cancer [40]. On the other hand, Min et al. showed that *HOTAIR* rs12826786 and rs920778 had an increased thyroid cancer risk, whereas rs7958904, rs4759314, rs874945, and rs189963 did not correlate with increased thyroid cancer risk [41].

#### 3.2.4. SNPs of *XRCC1* Gene

The X-ray repair cross-complementing group 1 (XRCC1) proteins have a central role in the base excision repair, an essential DNA repair pathway, thus preserving the genome stability [42]. In the past years, the role of the *XRCC1* Arg194Trp polymorphism in the development of DTC has been investigated. A meta-analysis published in 2016 by Zhao JZ et al. pointed out the *XRCC1* Arg194Trp polymorphism had an increased thyroid cancer risk in the Caucasian population [43]. On the other hand, in a more recent study published by Liu SY that also investigated *XRCC1* Arg194Trp polymorphism and its association with susceptibility to thyroid cancer, the C allele of *XRCC1* had an 18% significantly decreased risk of thyroid cancer in Chinese people, but without any association among Caucasians [44]. 

### 3.3. SNPs of the VDR Gene

Low vitamin D levels were correlated with an increased risk of advanced papillary thyroid cancer in several studies, being associated with local or distant metastasis and having a potential prognostic impact [45,46,47]. Vitamin D receptor (*VDR*) polymorphisms might increase the risk of several types of cancer (breast, ovarian, colorectal) [48,49,50]. 

Regarding thyroid cancer, Beysel et al. published a case-control study in 2018 and found that the *VDR* gene FokI (rs2228570) CT/TT genotype had an increased risk of PTC, whereas the FokI TT genotype usually presented with increased tumor diameter (T3 and T4), advanced stage (III/IV), and extra-thyroidal invasion. Moreover, the FokI CT/TT or TT genotypes were associated with lymph node metastasis, multifocality, and tumors >10 mm [51]. Another study conducted on Romanian patients with DTC investigated the correlation between vitamin D levels, *VDR* gene polymorphisms, clinical findings, and histopathological traits and found that vitamin D levels were significantly lower in patients with DTC and that FokI polymorphisms were frequently encountered in patients with DTC. Moreover, the Ff genotype was associated with more aggressive forms [52]. 

These studies showed promising results and future research should focus on studying in a more detailed manner the opportunities of using FokI as a poor prognostic factor in DTC.

### 3.4. SNPs of Extracellular Matrix Genes with Roles in Cellular Proliferation and Differentiation

#### 3.4.1. SNPs of *SPARC* and *SPP1* Gene

Matricellular proteins such as secreted phosphoprotein 1 (SPP1) and secreted protein acidic and rich in cysteine (SPARC) are involved in preserving cellular stability and structure [53]. The aberrant expression of these proteins might lead to several types of cancer progression or metastases [54,55]. Su X et al. noted that three loci, rs1054204, rs3210714, and rs3549 in *SPARC* and rs4754 in *SPP1*, individually or combined, are involved in PTC susceptibility, either decreasing or increasing the risk of developing the disease [56]. Currently, data regarding genetic variants of matricellular proteins in thyroid cancer are scarce, therefore there is great potential to explore this research area through future studies.

#### 3.4.2. SNPs of *MMP-9* Gene

Matrix metalloproteinases, also knowns as matrixins, are calcium-dependent and zinc-containing endoproteinases, which are capable of degrading numerous extracellular matrix proteins and processing several types of bioactive molecules, having an important part in processes such as cell proliferation, adhesion/dispersion, angiogenesis, and apoptosis [57]. Matrix metalloproteinase-9 (MMP-9) is one of the most important and complex MMPs, with implications in inflammatory activity, immune response, activation of tumor growth factor β in cancer progression, and resistance of tumor cells, which has a strong application as a cancer growth, invasion, and metastasis mediator [58]. MMP-9 has a physiologically low-level expression but is overexpressed in several types of cancers [58,59]. 

Serum MMP-9 levels measured using an immunometric assay were evaluated in subjects with PTC in a case-control study that concluded that although serum MMP-9 is not helpful in the diagnosis of PTC, a high pre-surgical level might be a prognostic factor that could dictate the treatment aggressiveness [60]. In a recent study that investigated the role of *MMP-9* promoter −1562C/T functional SNP in the risk of developing PTC, the T allele was found significantly more frequently in subjects with PTC (17.5% vs. 10.1%, *p* = 0.019) [61]. PTC had an increased risk of developing in subjects with the CT or CT + TT genotype. Although *MMP-9* promoter SNP seems to increase the susceptibility of developing PTC, it was not associated with tumor histological subtype, invasion, or tumor stage [61]. Nevertheless, despite these promising results that strongly suggest a role for *MMP-9* SNPs in thyroid carcinogenesis, future research is still needed on larger populations.

#### 3.4.3. SNPs of *NRG1* Gene

The neuregulin 1 (*NRG1*) gene encodes a glycoprotein that mediates cell–cell signaling. It is produced in multiple isoforms and plays an important part in the proliferation, survival, and differentiation of various system organs [62]. Several studies demonstrated an association between *NRG1* rs2439302 and PTC [62,63,64]. Moreover, a recent meta-analysis consolidated the previous results stating that the SNP rs2439302 variants of the gene encoding *NRG1* carry a high thyroid cancer risk, especially in Chinese populations compared to Japanese populations or populations from the United States of America [65].

### 3.5. SNPs of Genes Involved in Thyroid Morphogenesis and Function

#### 3.5.1. SNPs of *FOXE1* Gene

The Forkhead factor E1 (*FOXE1*) gene is a thyroid transcription factor and a member of the forkhead/winged-helix family with a crucial role in thyroid morphogenesis. It is also a key factor in follicular cell proliferation and differentiation, thus playing an important part in thyroid tumorigenesis [66]. *FOXE1* rs965513 SNP offered an increased risk for DTC in a German population studied in a case-control study from 2014 conducted by Penna-Martinez et al., including in advanced tumor staging [67]. A meta-analysis published in 2018 by Chen YH et al. that combined the results of 15 studies found that common variants of *FOXE1* rs965513, rs944289, and rs1867277 were risk factors for increased susceptibility to developing DTC [68]. However, the previously mentioned study was conducted mainly on Caucasians, with a small sample of Asian studies. Recently, a case-control study conducted in Iran on 81 patients with thyroid cancer and 165 controls found that *FOXE1* polymorphism (rs1867277) could be used as a biomarker for thyroid cancer diagnosis, being found in 32.1% (homozygous GG) and 18.5% (homozygous AA) of thyroid cancer patients [69].

#### 3.5.2. SNPs of *TSH-β* Gene

The thyroid-stimulating hormone (TSH) has two distinct subunits, namely the alpha subunit (TSH-α), which is common to all glycoprotein hormones, and the beta subunit (TSH-β), which ensures a normal functioning TSH [70,71]. Given that the TSH level influences thyroid function, genetic changes in the synthesis pathways of TSH might lead to thyroid diseases, including thyroid cancer. Mutations in the *TSH-β* gene might be linked to several types of thyroid dysfunctions, such as congenital central hypothyroidism or severe isolated TSH deficiency [72,73]. However, the *TSH-β* gene has an unclear role in the pathogenesis of thyroid cancer with little data in the literature. SNPs in the *TSH-β* gene and their role in the development and evolution of thyroid cancer have been investigated quite recently. A prospective case-control study conducted on 507 Saudi patients with DTC pointed out that *TSH-β* gene polymorphism (rs201857310) is associated with the disease and might have a role in its pathogenesis in this population [74]. Nevertheless, larger studies are required to verify this finding, expanding the research towards diverse ethnicities and analyzing the exposure to distinct environmental factors as well.

### 3.6. SNPs of Genes Involved in Folate Metabolism

Folate metabolism is known to be implicated in various processes such as synthesis, methylation, and DNA repair, with some genes such as methylenetetrahydrofolate reductase (*MTHFR*), methionine synthase (*MTR*), reduced folate carrier 1 (*RFC1*), and cystathionine β-synthase (*CßS*) having an important part in regulating this metabolism [75]. Genetic alterations in these genes could potentially lead to genetic instability, therefore increasing the risk of carcinogenesis. 

Several studies in the literature state that genetic SNPs involved in folate metabolism could increase the likelihood of developing several types of cancer [76,77,78,79]. A study published in 2019 by Zara-Lopes et al. investigated the associations between *MTHFR* 677C > T, *MTR* 2756A > G, and *RFC1* 80A > G polymorphisms and thyroid cancer, also evaluating the association between these SNPs, risk factors, and clinico-histopathological features [75]. The study revealed an association between *MTHFR* 677C > 7 and thyroid cancer under the dominant, codominant, and recessive models. Furthermore, *RFC1* 80A > G was also associated with thyroid cancer, but only under the recessive model, whereas *MTR* 2756A > G showed an association with tumor size and aggressive behavior [75]. 

Based on these results, larger studies focusing on *MTHFR* 677C > 7 and *MTR* 2756A > G SNPs should be conducted on different populations to fully assess the potential of these SNPs as prognosis factors.

### 3.7. SNPs of Genes Involved in Inflammation

Nowadays, inflammation is considered a hallmark of cancer. The role of inflammation in cancer has been investigated by numerous researchers and was linked to cancer occurrence, metastasis, or treatment resistance. Therefore, targeting genes involved in the inflammatory process might have a great impact on cancer management. 

Cytokines, especially several interleukins, play a critical role in the interaction between immune and non-immune cells in the tumor microenvironment, having implications for cancer development and progression [80]. Other molecules, such as nitric oxide, have an anti-inflammatory effect in physiological conditions, but can develop pro-inflammatory action in pathological situations [81]. In the following section, we aimed to briefly summarize the most recent results regarding SNPs in inflammation-related genes and thyroid cancer.

#### 3.7.1. SNPs of *IL-10* Gene

It is widely known that cytokines influence the activation, growth, and differentiation of various cells, also having a potential role in cancer occurrence. Tumor necrosis factor α (TNF-α) and interleukin-6 (IL-6) have a pivotal role in initiating the inflammatory response. Moreover, they are secreted by several types of cancer to promote tumoral growth [82]. On the other hand, interleukin-10 (IL-10) is an immunosuppressive cytokine that can stimulate cancer growth by helping the malignant cells escape the immune response [83]. In 2014, Cil et al. published a case-control study conducted on 190 patients with thyroid cancer and 216 controls which investigated the association between *TNF-α* G-308A, *IL-6* G-174C, and *IL-10* G-1082A SNP and clinico-biochemical features of PTC [84]. They found that SNPs of *TNF-α* G-308A and *IL-6* G-174C had no impact on the risk of developing PTC. However, the frequency of the *IL-10* 1082-G allele was higher in the PTC group. Moreover, subjects with the *IL-10* G-1082 GG genotype had an increased risk of developing PTC compared to the AA genotype, concluding that SNP in the *IL-10* gene might have an important role in the pathogenesis of PTC [84].

#### 3.7.2. SNPs of *IL-1* Gene

The interleukin-1 (IL-1) system encompasses different cytokines that intervene in both innate inflammation and immune responses by acting upon two different subtypes of receptors: IL-1 receptor type 1 (IL1R1) and IL-1 receptor type 2 (IL1R2) [85]. Their genetic polymorphism seems to be linked to various autoimmune diseases, and sparsely, with inconclusive results, with PTC [86,87,88,89]. Xiong et al. published their results in 2019 after investigating the role of the *IL1R1* and *IL1R2* SNPs in thyroid cancer. They found that rs3917225 in *IL1R1* as well as rs2072472 and rs11674595 in *IL1R2* were linked to susceptibility to thyroid cancer [90].

Interleukin 1 alpha (IL1A) and interleukin 1 beta (IL1B) belong to the IL-1 protein cluster and are involved in chronic inflammation, as well as in cancer development [85]. In their paper, Li H et al. tried to determine whether *IL1A* and *IL1B* polymorphisms predispose to a higher risk of thyroid cancer. Twelve SNPs of *IL1A* and *IL1B* were examined, concluding that six of them (rs3783521, rs3783546, rs3783550, and rs1609682 for *IL1A* and rs3136558 and rs1143623 for *IL1B*) were linked to an increased susceptibility to thyroid cancer development. *IL1B* polymorphisms were associated with an increased thyroid cancer risk after the fifth decade of life (>48 years old), whereas *IL1A* polymorphisms and thyroid cancer susceptibility were linked to age below this threshold [91].

#### 3.7.3. SNPs of *NOS3* Gene

Nitric oxide is a radical that acts as a biological mediator in various processes in the body, being involved both at the level of macrovascularization and microvascularization. At the level of macrovascularization, nitric oxide’s function consists of suppressing inflammation and cell adhesion, inhibiting the thrombosis process, and improving blood flow. In terms of microvasculature, nitric oxide at this level promotes angiogenesis [92]. Nitric oxide has also been associated with modulating neuronal activity as well as with various types of cancer (breast cancer, melanoma metastases) [92]. Genetic variations in the nitric oxide synthase 3 (*NOS3*) gene can alter plasma nitric oxide concentrations while increasing oxygen free radical production [84]. Increased oxygen-free radicals can lead to oxidative stress with damage to DNA and RNA, increasing the risk of cancer [92,93]. 

As for its role in thyroid cancer, SNPs of the *NOS3* gene were studied in an observational case-control study conducted on a Brazilian population of 31 patients diagnosed with PTC, who were operated on and treated with radioiodine (I131), and 81 healthy controls [94]. There was a significant genotype difference between the PTC group and the control group (*p* = 0.001). Furthermore, the BB genotype was considered a protective factor for PTC (*p* = 0.001), whereas the presence of the A allele appeared to be a risk factor (*p* = 0.001). The results also suggested that the polymorphism of the *NOS3* gene in intron 4 might increase the susceptibility for papillary thyroid carcinoma, though without detecting any association with patients’ clinical and biological characteristics [94]. There is very little data in the literature regarding genetic variations in the *NOS3* gene, therefore this topic should be addressed in future research considering the increased risk of malignancy that SNPs in this gene might be associated with. 

### 3.8. SNP in lncRNAs

Approximately 75% of the human genome is transcribed in RNAs, with less than 2% of the genes being translated into proteins, meaning that approximately 98% of RNAs are involved at the transcriptional levels and are composed of non-coding RNAs (ncRNAs). These ncRNAs are classified as short ncRNAs or long non-coding RNAs (lncRNAs) [95,96]. The miRNAs are small, non-coding RNAs that regulate gene expression, and their dysfunction alters the expression of oncogenic or tumor-suppressor target genes [97]. Recently, Khan et al. demonstrated a strong association between miRNA-149 (*MIR149*) gene SNP rs2292832 and thyroid cancer, specifically the mutation T > C (*p* = 0.0004) [98]. Furthermore, Khan et al. observed that miRNA-34b/c (*MIR34B*) gene SNP rs4938723 had a strong association with thyroid cancer occurrence [99]. 

As for lncRNAs, they have received great cancer research interest in the past years. Numerous studies have demonstrated that these molecules are involved in various physiological and pathological processes, including carcinogenesis or metastasis. These processes play a critical role in the regulation of gene expression, chromatin remodeling, transcription, and post-transcriptional processes. At the same time, certain lncRNAs can play the role of tumor suppressors or oncogenes in various cancers [96]. 

Regarding thyroid cancer, in 2016, Yang et al. found 675 differentially expressed lncRNAs in PTC, out of which 312 were upregulated and 363 downregulated [100]. Some important lncRNAs studied over the years in thyroid cancer include Nuclear Paraspeckle Assembly Transcript 1 (*NEAT1*), *HOTAIR*, GAS8 Antisense RNA 1 (*GAS8-AS1*), Papillary Thyroid Carcinoma Susceptibility Candidate 3 (*PTCSC3*), Maternally Expressed 3 (*MEG3*), BRAF-Activated Non-Protein Coding RNA (*BANCR*), cancer susceptibility candidate 2 (*CASC2*), and Metastasis Associated Lung Adenocarcinoma Transcript 1 (*MALAT1*) [101]. As for investigating lncRNAs SNPs and thyroid cancer, several studies have investigated possible links between lncRNAs SNPs and PTC, establishing, for example, that lncRNAs *BANCR*, *PTCSC3*, and *MEG3* are downregulated and have tumor suppressor roles in thyroid cancer, whereas lncRNAs CDKN2B Antisense RNA 1 (*ANRIL*), *MALAT1*, *HOTAIR*, and *HIT000218960* are upregulated and function as oncogenes [102,103,104,105,106,107,108,109]. Huang et al. investigated the effects of lncRNA *CASC2* in PTC. They found that the overexpression of *CASC2* promoted inhibition of carcinogenesis in PTC, suggesting that *CASC2* might have a potential role as a prognostic or therapeutic biomarker [110]. A possible novel therapeutic biomarker could be the lncRNA Copy Number Amplified Long noncoding RNA in Papillary Thyroid Cancer 1 (*CNALPTC1*), which was named and investigated by Chen et al. in 2018. They found that *CNALPTC1* was upregulated in PTC and its overexpression was associated with aggressive behavior [111].

lncRNAs SNPs represent a new and exciting cancer research area. Several new studies have investigated lncRNAs SNPs and various results have been reported in recent years, hinting at their possible role as biomarkers of diagnosis and prognosis in thyroid cancer.

A recent study conducted by Maurei-Milan et al. investigated the link between lncRNAs *ANRIL* SNP, which plays the role of a tumor suppressor gene, and PTC, also investigating the link with various clinical features of this pathology. They determined *ANRIL* SNPs in rs11333048, rs4977574, rs4977574, rs1333040, and rs10757274 in 134 PTC patients and 155 controls. The results showed that the AAAC haplotype had a protective effect from PTC, whereas the CAAC and CAGT haplotypes were associated with increased cancer risk. Moreover, the rs1333048 CC variant was found more frequently in tumors >1 cm, whereas the rs4977574 AC variant was found predominantly in smaller tumor sizes. Furthermore, SNPs in rs10757274 and rs1333040 had a lower chance of advanced disease [95]. 

Chen et al. concluded that lncRNA RNA Polymerase II, I And III Subunit E (*POLR2E*) rs3787916 was associated with increased cancer risk [112]. Wen et al. investigated the association between *MALAT1* SNP and thyroid cancer susceptibility and found that *MALAT1* SNP rs619586 was a protective factor of PTC (*p* = 0.017) [113]. In 2022, Wang et al. published their results following an investigation between SNP rs8101923 within terminal differentiation-induced lncRNA (*TINCR*) and the risk of PTC and found that rs8101923 was a risk factor for the pathogenesis of PTC, with the G allele having a significantly associated higher risk of PTC [114].

There is still a very large spectrum for future research of lncRNAs SNPs that might have a great prognostic and/or therapeutic impact in DTC.

## 4. Future Perspectives

Thyroid cancer, like all other types of cancer, is a complex disease where numerous genetic alterations take place. In the past years, genetic research on cancer has been an important topic for the scientific world. The results of the already published studies or the upcoming research may shed some light on the underlying biological pathological processes involved in this disease. These data could provide a possibility of developing therapeutic targets and biomarkers that could be used in preventing thyroid cancer or establishing a personalized approach to diagnosis and treatment, thus increasing the chance for a better prognosis.

As emphasized in this review, several SNPs in various genes were studied with regards to their association with susceptibility, morpho-pathological traits, and prognosis in DTC (Table 1). Furthermore, lncRNAs and their role as biomarkers for tumor diagnosis, prognosis, and treatment have received great interest and are expected to become one of the main targets in gene-targeted therapy. However, this domain is still unclear, and further studies are needed to overcome the great challenges that come with researching lncRNAs.

Nevertheless, there are still questions to be raised concerning the exact biological mechanisms involved with the genetic variants and their effects, although considering the numerous statistically significant results found across the literature is a strong argument for their implication in thyroid carcinogenesis, and without doubt, there are many other SNPs that could impact not only the genetic predisposition to thyroid cancer but also cancer’s clinico-histopathological behavior.

Some directions that future research should follow are to include larger cohorts, to take into consideration in a greater manner the ethnicity and environmental factors, and to define better study designs to increase the chance of fully understanding their effect on thyroid oncogenesis. Another main research purpose should be to define in detail the cancer phenotype, such as tumor histological subtype, behavior, aggressive clinico-histopathological traits, and the association with specific genetic variants. Therefore, by analyzing in depth the phenotype of patients with thyroid cancer (such as ethnicity, environmental factors, and clinico-histopathological characteristics of the tumor) and the genetic variants, a better understanding of small associations and gene–environment interactions would be expected.

## 5. Conclusions

In the last decades, important progress has been made regarding the genetic analysis of thyroid cancer. The knowledge we gather from genetic studies plays an undeniable role in understanding the pathogenesis of thyroid cancer and might help in developing new diagnostic and prognostic biomarkers. Furthermore, analyzing the genotype and the presence of SNPs of several genes could help clinicians tailor personalized management as well as inform individuals who are at risk of developing thyroid cancer. Nevertheless, the existing literature data on genetic polymorphisms must be enriched with further studies, ideally consisting of larger cohorts, also considering the subjects’ ethnicities and environment for a better understanding of the oncogenic mechanisms involved in thyroid cancer.

## Figures and Tables

**Table 1:** Summary of genetic variations associated with thyroid cancer susceptibility and clinico-histopathological traits.

Gene	Category	SNP	Main Results	Reference
*RET*	Protein-coding	S836S	Overexpressed in patients with PTC	[23]
		G691S	Identified in tumors > 10 mm	
		S904S		
		rs17028, rs1799939, rs1800858, rs1800860, rs2075912, rs2565200, rs2742240	Haplotype CAAGCGT: increased risk for distant metastases rs1799939 AG/AG plus AA genotypes: increased risk for DTC without concomitant thyroid benign disorders	[24]
		rs1799939	Increased thyroid cancer susceptibility	[25]
*BAX*	Protein-coding	−248 G > A	Significant genotypic difference between PTC and healthy subjects Association with PTC G allele: protective factor for PTC	[32]
*TSH-β*	Protein-coding	rs201857310	Strongly associated with thyroid cancer	[74]
*VDR*	Protein-coding	rs2228570	CT/TT/Ff genotype: increased PTC susceptibility TT/Ff genotype: tumor diameter T3 and T4, stage III/IV, and extra-thyroidal invasion T allele: increased risk of tumor-node-metastasis, greater tumor diameter, multifocality, and extra-thyroidal invasion	[51,52]
*SPARC*	Protein-coding	rs1054204, rs3210714, rs3549	Reduced PTC risk	[56]
*SPP1*	Protein-coding	rs4754	Increased PTC risk	[56]
*XRCC1*	Protein-coding	Arg194Trp	Increased thyroid cancer risk	[43]
			C allele: decreased thyroid cancer risk	[44]
*NRG1*	Protein-coding	rs2439302	Role in PTC predisposition G allele: high expression of multiple NRG1 isoforms in unaffected thyroid tissue	[62]
			Increased thyroid cancer risk	[63,64,65]
*HOTAIR*	Non-coding	rs1899663	No association with clinicopathological traits of thyroid cancer	[40]
			No association with increased thyroid cancer risk	[41]
		rs7958904, rs4759314, rs874945	No association with increased thyroid cancer risk	[41]
		rs12826786, rs920778	Increased thyroid cancer risk	[41]
*TP53*	Protein-coding	rs1042522	CC genotype: protection against PTC Associated with tumors > 10 mm and with vascular invasion	[37]
*p21*	Protein-coding	rs10559234, rs1801270	No association with increased thyroid cancer risk	[37]
*FOXE1*	Protein-coding	rs965513	Increased susceptibility for DTC	[67,68]
		rs944289, rs1867277		[68,69]
*IL-10*	Protein-coding	G-1082A	G allele: more frequent in PTC patients compared to controls GG genotype: increased risk of developing thyroid cancer compared to AA genotype	[84]
*IL1R1*	Protein-coding	rs3917225	Increased susceptibility for thyroid cancer	[90]
*IL1R2*	Protein-coding	rs2072472, rs11674595	Increased susceptibility for thyroid cancer	[90]
*IL1A*	Protein-coding	rs3783521, rs3783546, rs3783550, rs1609682	Increased susceptibility for thyroid cancer	[91]
*IL1B*	Protein-coding	rs3136558, rs1143623	Increased susceptibility for thyroid cancer	[91]
*MET*	Protein-coding	rs1621	AG genotype: increased risk for PTC Not associated with clinicopathological traits of thyroid cancer	[28]
*NOS3*	Protein-coding	Intron4	BB genotype: protective factor for PTC A allele: risk factor for PTC	[94]
*MTHFR*	Protein-coding	677C > T	Increased risk for thyroid cancer	[75]
*MTR*	Protein-coding	2756A > G	Associated with histopathological traits such as tumor extent and aggressive behavior	[75]
*RFC1*	Protein-coding	80A > G	Increased risk for thyroid cancer	[75]
*MMP-9*	Protein-coding	−1562C/T	CT or CT + TT genotype: increased risk for PTC	[61]
*MIR149*	Non-coding	rs2292832	Mutation T > C was strongly associated with thyroid cancer	[98]
*MIR34B*	Non-coding	rs4938723	Increased risk for thyroid cancer	[99]
*ANRIL*	Non-coding	rs11333048, rs4977574, rs4977574, rs1333040, rs10757274	AAAC haplotype: protective effect from PTC CAAC, CAGT haplotypes: increased thyroid cancer risk rs1333048 CC variant: frequently in tumors > 1 cm s4977574 AC variant: frequently in smaller tumor sizes rs10757274, rs1333040: low risk of advanced disease	[95]
*MALAT1*	Non-coding	rs619586	Protective factor for PTC	[113]
*POLR2E*	Protein-coding	rs3787916	Increased risk for thyroid cancer	[112]
*TINCR*	Protein-coding	rs8101923	G allele: high risk for PTC	[114]

Abbreviations: RET, Rearranged during Transfection; BAX, B-cell lymphoma 2-associated X protein; TSH-β, thyroid-stimulating hormone beta subunit; VDR, vitamin D receptor; SPARC, secreted protein acidic and rich in cysteine; SPP1, secreted phosphoprotein 1; XRCC1, X-ray repair cross-complementing group 1; NRG1, neuregulin 1; HOTAIR, HOX Transcript Antisense RNA; TP53, tumor protein 53; FOXE1, Forkhead factor E1; IL-10, interleukin-10; IL1R1, interleukin-1 receptor type 1; IL1R2, interleukin-1 receptor type 2; IL1A, interleukin-1 alpha; IL1B, interleukin-1 beta; MET, mesenchymal-epithelial transition factor; NOS3, nitric oxide synthase 3; MTHFR, methylenetetrahydrofolate reductase; MTR, methionine synthase; RFC-1, reduced folate carrier 1; MMP-9, Matrix metalloproteinase-9; MIR149, miRNA-149; MIR34B, miRNA-34b; ANRIL, CDKN2B antisense RNA 1; MALAT1, Metastasis Associated Lung Adenocarcinoma Transcript 1; POLR2E, RNA polymerase II, I and III subunit E; TINCR, terminal differentiation-induced noncoding RNA; PTC, papillary thyroid cancer; DTC, differentiated thyroid cancer.

## Data Availability

Not applicable.
